# Conflicts of interest in translational research

**DOI:** 10.1186/1479-5876-2-28

**Published:** 2004-08-09

**Authors:** Malcolm R Parks, Mary L Disis

**Affiliations:** 1 Office of Research, University of Washington, Seattle, WA, USA; 2 Tumor Vaccine Group, University of Washington, Seattle, WA, USA

## Abstract

Translational research requires a team approach to scientific inquiry and product development. Translational research teams consist of basic and clinical scientists who can be members of both academic and industrial communities. The conception, pre-clinical testing, and clinical evaluation of a diagnostic or therapeutic approach demands an intense interaction between investigators with diverse backgrounds. As the barriers between industry and academia are removed, issues of potential conflict of interest become more complex. Translational researchers must become aware of the situations which constitute conflict of interest and understand how such conflicts can impact their research programs. Finally, the translational research community must participate in the dialogue ongoing in the public and private sectors and help shape the rules that will govern conflicts that arise during the evolution of their research programs.

## Introduction

By its nature, translational research crosses boundaries between basic science and clinical application. It places researchers in new contexts and ushers in a range of new contacts and relationships. Crossing these boundaries contributes directly to the creativity and social impact of translational medicine. But crossing these boundaries also gives rise to new and often conflicting obligations between researchers, their employers, and their industry sponsors. The public is rightfully concerned that the financial interests of researchers, their institutions, and their corporate sponsors may bias research. Yet history also teaches us that industry collaboration is often essential in realizing the promise of translational research. Industry collaboration has figured prominently in many translational research successes including recombinant growth hormone, angioplasty, stenting for coronary artery disease, and many new medications and diagnostic devices [1].

Translational researchers must, therefore, understand what financial conflicts of interest are and how they are managed. Their industry partners must understand the constraints placed on researchers by federal and university policies as well as state laws. Relevant policies in the United States include the regulations issued by the Public Health Service and published as part of the Code of Federal Regulations (42 CFR 50.601–50.607) and in the National Science Foundation Grant Policy Guide (Section 510) [2,3]. Laws governing the use of state-owned resources may also be relevant for those working at or with public universities.

## What triggers financial conflicts of interest?

Conflicts may arise whenever researchers' outside, personal financial interests have the potential to compromise an investigator's professional judgment and independence in the design, conduct, or publication of research. The most commonly regulated financial interests include consulting fees or compensation for personal services, equity or other ownership interests, royalties, and intellectual property rights. A researcher may, for example, receive consulting income or equity in exchange for service on a scientific advisory board of a company that then sponsors clinical research in her lab. Another researcher may be paid for talks to physician groups about an approved medication while simultaneously conducting research on potential off-label uses of the drug. The investigator or an immediate family member may hold stock in the research sponsor. These examples are all common cases and most institutions have relatively standardized ways of managing such common conflicts.

Greater challenges are created when the financial relationships between commercial interests and investigators are either ambiguous or complex. Ambiguity can result in a number of ways, but one of the most frequent occurs when investigators approach consulting as an extension of discussions among academic colleagues. Thinking that talking to a corporate representative is the same as talking to an academic colleague, for example, may lead the investigator to make inappropriate disclosures that compromise intellectual property rights or contractual obligations. Often it is not any one financial interest, but rather the combination of multiple financial interests that makes a situation unmanageable. It is extraordinarily difficult, for example, to manage situations in which an investigator is the founder of a startup company, an inventor on a patent licensed to the company, a consultant to the company, and the recipient of other government and industry grants for closely related research.

## How institutions manage potential conflicts of financial interest

Spurred by a combination of bad experiences and new regulations, most research institutions now have policies in place for the management of some aspects of personal financial interests in research. One strategy is simply to prohibit personal financial interests in research. The Association of American Universities, for example, advocates outright prohibition in cases involving research on human subjects unless there are "compelling circumstances" that justify an exception [4]. Prohibition forces financially interested researchers to either divest their interests or to remove themselves from the research.

Although effective, prohibitions are blunt tools and in our opinion should be used only as a last resort. We say this not only because prohibiting financial interests may leave any number of other equally biasing interests in place [5], but also because, when properly managed, financial interests may play a positive role in the development of a translational research program. Access to company resources and sharing investigator knowledge are often critical to timely translations of basic science to clinical practice. The complex research enterprise needed to develop clinical products is simply beyond the scope of what many investigators can achieve on their own in academic institutions.

Fortunately, there are usually less draconian alternatives to outright prohibition. These strategies seek to ensure the integrity of the research, guarantee public scrutiny and access, and, of course, to protect human participants [6]. One of most common is to assign key research activities such as recruiting, consenting, and data analysis to team members who have no financial stake in the results. Multi-center designs ensure that the biases of any one investigator are less likely to influence the final results. Independent data safety monitoring boards or other oversight committees may also check the influence of personal financial interests. So, too, will requirements to disclose financial interests to publishers, conference organizers, and institutional review boards.

Research integrity is further protected by a vigilant stance regarding publication restrictions. Industry partners have a legitimate interest in protecting proprietary information, but this can usually be honored by providing a short period for review prior to submitting a manuscript for publication. No contract or agreement, however, should give the sponsor the right to control publication. Work that requires such control is more appropriately done in industry rather than in academic laboratories. The close attention to publication restrictions is particularly important when a researcher may have a student working on an industry sponsored project. Junior and student scientists working in a research program, who may not have any relationship with a company, must be able to have freedom in pursuing aspects of projects outside the bounds of the research agreement and publishing data in a timely fashion.

Translational research that results in the development of a new company presents particular opportunities and challenges. Because of the importance of small companies to economic growth, public research universities often view the number of university-related startups as an index of their contribution to the state economy. More specific institutional interests are created when universities take equity in startups through licensing agreements. Unlike more established firms, start-up companies are often highly dependent on obtaining favorable research outcomes from a particular project. In many cases, prohibition may be the only way to manage the tangle of institutional and individual interests than can result in this situation. Universities may create "firewalls" between the management of equity and researchers [7]. They may require divestiture or bar researchers from receiving grants back from companies to which their inventions have been licensed. Consulting and other company contacts may be restricted when the investigator or the university has a significant financial interest in the startup. Although universities are still coming to grips with their own institutional financial interests, there is an emerging consensus that they should not conduct clinical research on their own inventions unless a strong case for locating the research at the university can be made on clinical grounds [8,9]. For novel biologic therapies, the investigator who invented the approach may be ideally suited to complete the clinical translation. The failure of many novel agents to demonstrate activity in Phase I may be linked to a disconnect between the scientist developing the agent and the physician running the clinical trial. Translational research is defined as that person being one and the same.

Recently, the American Association of Medical Colleges published suggested guidelines for the management of conflict of interest based on input from a number of medical schools [10]. Such documents not only encourage dialogue concerning these issues, but also provide some guidance for individual institutions establishing their internal policies.

## How financial conflicts of interest can affect your research program

Unrecognized and unmanaged conflicts of financial interest represent major risk factors for programs of translational research. Even when properly disclosed and managed, however, outside financial interests may limit your research activities in a variety of ways, ranging from mild to severe. If nothing else, time and staff resources must be allocated to institutional and extra-institutional review processes. Because approval is required before funding is released or human subjects are enrolled, the pace of research is slowed by outside financial interests.

Scientists with personal financial interests in the research will usually find themselves barred from participating in sensitive research activities, especially those involving direct contact with human subjects. This increases the costs of research as additional staff or consultants are hired to do the work that the financially interested party would have otherwise performed. But even this strategy may prove difficult if key staff or alternative expert consultants also have financial interests. There is a tipping point beyond which so many potential team members are financially involved that the interests simply can not be managed and outright prohibition becomes the only option.

In the most extreme circumstances it is possible for researchers to research themselves out of a job. Their personal financial interests in a research sponsor may be so great or so complex that their employers are unable to accept further funding from that sponsor. A line of translational research may simply end for a researcher when he or she is barred from carrying the work into the clinical arena as a result of individual or institutional financial interests. This can occur even when the researcher is not actively seeking financial gain. Early basic science work, for example, may lead to an invention that the university decides to patent and license. In most universities the researcher is entitled to a share of whatever revenue is produced. The researcher now has a personal financial interest that requires management and may disqualify her or him from participation in later clinical work designed to translate the basic science into practice. This is an extreme case, of course, but it does happen and it illustrates the often unseen and unintended implications of how financial interests are usually managed.

## Dealing intelligently with financial interests in research

Public concern about personal and institutional interests in research is not going to go away. Nor is the need for industry collaboration in translational research. Indeed the need for collaborations between industry and academia is only going to grow as we move beyond a sequential "bench to bedside" model to acknowledge the benefits of combining clinical and basic biological studies [11]. If financial interests can not be avoided, we can at least be more thoughtful about how we manage them-as individual researchers, as industry collaborators, and as academic research institutions.

Individual investigators should recognize that disclosure of personal financial interests, while perhaps uncomfortable, is vital to continued public confidence in science. They must also balance their wish for personal gain against the additional oversight and management of their activities that will inevitably result. They should recognize that some financial interests are more easily managed than others. Consulting income paid as cash, for example, is much more easily managed than consulting income paid with equity in the company. The latter creates a long-term financial interest and may imply management influence. The understandable desire to "share the wealth" with team members by assisting them in obtaining outside financial interests of their own backfires when it then becomes necessary to remove them from the tasks that they were hired to do in the first place.

The decision to create a company in order to further a translational research agenda is appealing, often appropriate, but always more complex than researchers typically appreciate. It is a risky conceit to believe that one's success in obtaining research grants and managing a research team is adequate preparation to launch a business. This is borne out by the fact that technologies licensed to companies founded by faculty inventors are generally less successful than those licensed to companies with non-academic founders [12]. Even when the researcher has the requisite business experience, however, company formation may result in profound conflicts of commitment, inappropriate use of university resources in support of the company, and confusion over intellectual property. These difficulties combine to increase costs and slow, if not block, progress on translational research efforts.

In spite of a growing entrepreneurial spirit within academia, the culture gap between industry and academia remains large. Industries seeking to partner with academic researchers must understand that universities are not simply laboratories for hire. They should not assume that they will own or control publication of the research they support. Universities are not set up to guarantee the same level of security and secrecy than can be obtained in industry laboratories. The open character of universities is in fact an asset- it is the foundation on which the creative engine rests. Finally it is useful to appreciate that "indirect costs" are real costs for universities. Indeed, even at the full rate, universities subsidize research contracts [13]. Efforts to avoid indirect costs, like efforts to negotiate contracts that contain publication restrictions and overreaching intellectual property clauses, ultimately have the effect of disrupting working relationships and slowing the pace of research. By the same token, industry collaborators should recognize that efforts to "build relationships" with academic researchers and their staff by providing extra financial incentives and benefits are often counterproductive, not only because they fail to buy loyalty, but also because they create unacceptable financial conflicts of interest for academic personnel.

Universities, too, have much to learn about managing financial interests and collaborating creatively with their industry partners. Although a consensus regarding institutional conflicts of interest is emerging, universities still have much to do in terms of implementation, particularly with regard to credible external review of clinical research opportunities in which the institution holds a financial interest. Managing or avoiding institutional conflicts of interest will also require a more realistic view of technology transfer opportunities. Although "big hits" do occur, they occur only rarely and revenue from technology transfer operations is typically only a tiny fraction of revenue from sponsored research. Universities run the risk of disrupting their larger research mission by overly aggressive efforts to capture and commercialize intellectual property. Open publication and teaching should always be the most significant "knowledge transfer" functions of a research university.

The increasing complexity of translational research also challenges universities to devise new kinds of collaboration with industry. Mankoff and her colleagues, for example, recently proposed the creation of centers that obtain revenue from existing therapies, while simultaneously providing material for biological studies and supporting experimental therapies [11]. Before these more complex collaborations can occur, however, there is much work to be done to simplify material transfer practices between laboratories and to create more straightforward ways for facilities and personnel to be shared.

Individual researchers, industries, and universities have much to learn about the management of financial interests. Given the potential for financial interests to disrupt or even end programs of translational research, we believe that all parties would benefit from greater attention and greater creativity in the management of conflicts of interest. A dialogue must be established between the individual investigator, industry, academic institutions, and the public to define the issues and develop rational solutions. Such a dialogue could be initiated by the development of a focus on such topics at national meetings, by individual organizations whose membership is involved in translational research developing interdisciplinary panels to discuss the issues and attempt to develop guidelines, and by an integration of conflict of interest topics in the training of junior scientists. Solutions should enhance, not impede, translational research.
